# Supplementary material for: DrugReasoner: Interpretable drug approval prediction with a reasoning-augmented language model
Source: PLoS One. 2026 Feb 19;21(2):e0342940. doi: 10.1371/journal.pone.0342940 (PMC12919791; doi:10.1371/journal.pone.0342940)
Supplement: S3 File — System instructions and an example user prompt provided to DrugReasoner. (DOCX) [file pone.0342940.s003.docx]

Table 1. System prompt used in the training and inference.

| You are a chemist specializing in drug discovery and molecular modeling. Your role is to evaluate the drug-likeness and viability of a given chemical compound as a potential drug candidate, using computational descriptors derived from RDKit.  For each compound, you are provided with:  - Molecular descriptors and properties calculated via RDKit.  - The RDKit-derived descriptors and properties of the five most similar approved and five most similar unapproved small molecules (based on structural similarity).  Follow these steps:  1. Analyze the compound's physicochemical properties.  2. Compare the compound with its most similar approved and unapproved counterparts, highlighting key similarities and differences that may influence drug-likeness or viability.  3. Think step by step and use your knowledge of molecular approval criteria and the patterns observed in both approved and unapproved compounds to explain your reasoning clearly and scientifically.  Based on your analysis, label the compound as either:  - approved  - unapproved  Then assign a confidence score between 0 and 1, where:  - A score near 1.0 indicates high confidence in your prediction (clear, strong evidence).  - A score near 0.0 indicates high uncertainty (ambiguous, conflicting, or weak evidence).  Respond in the following format:  <think>  (Your step-by-step scientific reasoning)  </think>  <label>  (approved or unapproved)  </label>  <score>  een 0 and 1)  </score>  (Numeric confidence score between 0 and 1) |
| --- |

Table 2. Input and reasoning samples in the training process.

| Compound's RDKit Properties:{'Molecular Weight': 385.42, 'LogP': 1.3, 'Molecular Refractivity': 101.59, 'TPSA': 98.57, 'Hydrogen Bond_Donors': 0, 'Hydrogen_Bond Acceptors': 7, 'Rotatable Bonds': 5, 'Chiral Centers': 1, 'Heavy Atoms': 28, 'Formal Charge': 0, 'Total Rings': 3, 'Structural Alerts': 'None'}              Five Most Similar Approved Compounds (RDKit): [{'Molecular Weight': 398.55, 'LogP': 2.5, 'Molecular Refractivity': 106.21, 'TPSA': 101.57, 'Hydrogen Bond_Donors': 2, 'Hydrogen_Bond Acceptors': 8, 'Rotatable Bonds': 10, 'Chiral Centers': 0, 'Heavy Atoms': 26, 'Formal Charge': 0, 'Total Rings': 2, 'Structural Alerts': ['aldehyde', 'disulphide']}, {'Molecular Weight': 563.67, 'LogP': 6.12, 'Molecular Refractivity': 150.63, 'TPSA': 110.21, 'Hydrogen Bond_Donors': 1, 'Hydrogen_Bond Acceptors': 6, 'Rotatable Bonds': 14, 'Chiral Centers': 4, 'Heavy Atoms': 39, 'Formal Charge': 0, 'Total Rings': 3, 'Structural Alerts': ['Aliphatic_long_chain', 'het-C-het_not_in_ring', 'phosphor']}, {'Molecular Weight': 215.29, 'LogP': 1.42, 'Molecular Refractivity': 58.4, 'TPSA': 69.39, 'Hydrogen Bond_Donors': 1, 'Hydrogen_Bond Acceptors': 4, 'Rotatable Bonds': 9, 'Chiral Centers': 0, 'Heavy Atoms': 15, 'Formal Charge': 0, 'Total Rings': 0, 'Structural Alerts': ['Aliphatic_long_chain']}, {'Molecular Weight': 424.62, 'LogP': 5.12, 'Molecular Refractivity': 120.06, 'TPSA': 83.83, 'Hydrogen Bond_Donors': 2, 'Hydrogen_Bond Acceptors': 5, 'Rotatable Bonds': 16, 'Chiral Centers': 4, 'Heavy Atoms': 30, 'Formal Charge': 0, 'Total Rings': 1, 'Structural Alerts': ['Aliphatic_long_chain', 'isolated_alkene']}, {'Molecular Weight': 575.69, 'LogP': 5.7, 'Molecular Refractivity': 156.91, 'TPSA': 115.73, 'Hydrogen Bond_Donors': 2, 'Hydrogen_Bond Acceptors': 7, 'Rotatable Bonds': 8, 'Chiral Centers': 0, 'Heavy Atoms': 41, 'Formal Charge': 0, 'Total Rings': 5, 'Structural Alerts': 'None'}]                Five Most Similar Unapproved Compounds (RDKit): [{'Molecular Weight': 427.41, 'LogP': 3.91, 'Molecular Refractivity': 116.14, 'TPSA': 94.83, 'Hydrogen Bond_Donors': 1, 'Hydrogen_Bond Acceptors': 7, 'Rotatable Bonds': 3, 'Chiral Centers': 0, 'Heavy Atoms': 32, 'Formal Charge': 0, 'Total Rings': 5, 'Structural Alerts': ['quinone_A(370)']}, {'Molecular Weight': 360.3, 'LogP': 0.9, 'Molecular Refractivity': 85.81, 'TPSA': 107.46, 'Hydrogen Bond_Donors': 1, 'Hydrogen_Bond Acceptors': 7, 'Rotatable Bonds': 3, 'Chiral Centers': 0, 'Heavy Atoms': 26, 'Formal Charge': 0, 'Total Rings': 3, 'Structural Alerts': 'None'}, {'Molecular Weight': 546.58, 'LogP': 4.86, 'Molecular Refractivity': 153.07, 'TPSA': 107.72, 'Hydrogen Bond_Donors': 1, 'Hydrogen_Bond Acceptors': 8, 'Rotatable Bonds': 4, 'Chiral Centers': 0, 'Heavy Atoms': 41, 'Formal Charge': 0, 'Total Rings': 7, 'Structural Alerts': 'None'}, {'Molecular Weight': 451.55, 'LogP': 1.9, 'Molecular Refractivity': 116.56, 'TPSA': 119.49, 'Hydrogen Bond_Donors': 2, 'Hydrogen_Bond Acceptors': 7, 'Rotatable Bonds': 11, 'Chiral Centers': 0, 'Heavy Atoms': 31, 'Formal Charge': 0, 'Total Rings': 3, 'Structural Alerts': ['Aliphatic_long_chain']}, {'Molecular Weight': 535.05, 'LogP': 2.11, 'Molecular Refractivity': 134.91, 'TPSA': 117.58, 'Hydrogen Bond_Donors': 2, 'Hydrogen_Bond Acceptors': 7, 'Rotatable Bonds': 7, 'Chiral Centers': 2, 'Heavy Atoms': 35, 'Formal Charge': 0, 'Total Rings': 4, 'Structural Alerts': 'None'}]  True_label:  unapproved  Response:  <think>  The given compound has a molecular weight of 385.42, logP of 1.3, and a relatively high TPSA of 98.57, which may indicate poor permeability across cell membranes. It has seven hydrogen bond acceptors but no hydrogen bond donors, suggesting it may have high potential for forming bonds with biological targets but lacks a donor group. The presence of three rotatable bonds and one chiral center indicates flexibility and a potential for stereoselectivity.  Among the approved molecules, the closest hit is the first one with a molecular weight of 398.55 and logP of 2.5, which shares similarities in molecular weight and number of rotatable bonds. However, the significant difference in logP and TPSA could be a deterrent for this compound.  The unapproved molecule with a molecular weight of 427.41 and logP of 3.91 shows a high degree of structural similarity, including the number of hydrogen bond acceptors and rotatable bonds. This molecule's structural alerts do not pose significant concerns, making it a strong candidate for similarity.  On the other hand, the molecule with a molecular weight of 360.3 and logP of 0.9 shares similarities in molecular weight and the absence of structural alerts but has a much lower logP and a different overall molecular structure.  The unapproved molecule with a molecular weight of 451.55 and logP of 1.9 also shows similarities in molecular weight and the presence of a structural alert (Aliphatic_long_chain), which might be less concerning than the quinone alert in the first unapproved compound.  The similarity between the compound and the unapproved molecule with a molecular weight of 427.41 suggests a high degree of conformational similarity and a likelihood of similar pharmacokinetic properties.  </think>  <label>  unapproved  </label>  <score>  0.87  </score>  Reasoning: The similarity between the compound and the unapproved molecule with a molecular weight of 427.41 suggests a high degree of conformational similarity and a likelihood of similar pharmacokinetic properties. The confidence score is 0.87 due to the significant structural similarities between the two molecules, particularly in the number of hydrogen bond acceptors and rotatable bonds. However, the absence of significant structural alerts in the unapproved molecule and the presence of a quinone alert in the first unapproved compound reduce the confidence score slightly.  Extracted_label:  unapproved  Score:  0.87 |
| --- |
