# Supplementary material for: DrugReasoner: Interpretable drug approval prediction with a reasoning-augmented language model
Source: PLoS One. 2026 Feb 19;21(2):e0342940. doi: 10.1371/journal.pone.0342940 (PMC12919791; doi:10.1371/journal.pone.0342940)
Supplement: S4 File — Performance comparison between baseline models, Ghose filter, QED, ChemAP, and DrugReasoner across validation, test, and external validation datasets. (DOCX) [file pone.0342940.s004.docx]

Table 1. Models’ performance on the validation set.

| Model | AUC | F1 | Recall | Specificity | Precision |
| --- | --- | --- | --- | --- | --- |
| Logistic regression | 0.696 | 0.675 | 0.628 | 0.764 | 0.728 |
| KNN | 0.699 | 0.691 | 0.673 | 0.724 | 0.71 |
| SVM | 0.692 | 0.652 | 0.575 | 0.809 | 0.751 |
| XGBoost | 0.707 | 0.69 | 0.65 | 0.764 | 0.735 |
| Ghose filter | 0.38 | 0.36 | 0.35 | 0.40 | 0.37 |
| QED | 0.47 | 0.52 | 0.56 | 0.38 | 0.48 |
| DrugReasoner | 0.732 | 0.729 | 0.721 | 0.742 | 0.738 |

KNN: K-nearest neighbors, SVM: Support vector machine, QED: Quantitative estimate of drug-likeness

Table 2. Models’ performance on the test set.

| Model | AUC | F1 | Recall | Specificity | Precision |
| --- | --- | --- | --- | --- | --- |
| Logistic regression | 0.672 | 0.657 | 0.631 | 0.712 | 0.686 |
| KNN | 0.716 | 0.713 | 0.707 | 0.726 | 0.719 |
| SVM | 0.672 | 0.635 | 0.573 | 0.77 | 0.713 |
| XGBoost | 0.732 | 0.718 | 0.684 | 0.779 | 0.755 |
| Ghose filter | 0.41 | 0.41 | 0.40 | 0.42 | 0.41 |
| QED | 0.47 | 0.52 | 0.57 | 0.37 | 0.48 |
| DrugReasoner | 0.725 | 0.718 | 0.702 | 0.748 | 0.735 |

KNN: K-nearest neighbors, SVM: Support vector machine, QED: Quantitative estimate of drug-likeness

Table 3. Models’ performance on the external dataset.

| Model | AUC | F1 | Recall | Specificity | Precision |
| --- | --- | --- | --- | --- | --- |
| Logistic regression | 0.559 | 0.211 | 0.118 | 1 | 1 |
| KNN | 0.618 | 0.381 | 0.235 | 1 | 1 |
| SVM | 0.529 | 0.111 | 0.059 | 1 | 1 |
| XGBoost | 0.588 | 0.3 | 0.176 | 1 | 1 |
| Ghose filter | 0.49 | 0.55 | 0.47 | 0.50 | 0.67 |
| QED | 0.61 | 0.59 | 0.47 | 0.75 | 0.80 |
| ChemAP | 0.64 | 0.643 | 0.529 | 0.75 | 0.818 |
| DrugReasoner | 0.728 | 0.774 | 0.706 | 0.75 | 0.857 |

KNN: K-nearest neighbors, SVM: Support vector machine, ChemAP: Chemical structure-based drug approval predictor, QED: Quantitative estimate of drug-likeness
