# Supplementary material for: DrugReasoner: Interpretable drug approval prediction with a reasoning-augmented language model
Source: PLoS One. 2026 Feb 19;21(2):e0342940. doi: 10.1371/journal.pone.0342940 (PMC12919791; doi:10.1371/journal.pone.0342940)
Supplement: S6 File — Performance metrics during ablation analysis. (DOCX) [file pone.0342940.s006.docx]

Table 1. Model’s performance when removing the comparative reasoning module.

| Set | AUC | F1 score | Recall | Specificity | Precision |
| --- | --- | --- | --- | --- | --- |
| Validation | 0.498 | 0.050 | 0.026 | 0.969 | 0.461 |
| Test | 0.500 | 0.026 | 0.013 | 0.987 | 0.5 |
| External | 0.559 | 0.210 | 0.118 | 1 | 1 |

Table 2. Model’s performance when removing the reasoning module from the pipeline (direct prediction using only MOLFORMER-extracted molecular features classified via XGBoost).

| Set | AUC | F1 score | Recall | Specificity | Precision |
| --- | --- | --- | --- | --- | --- |
| Validation | 0.760 | 0.759 | 0.752 | 0.769 | 0.766 |
| Test | 0.785 | 0.790 | 0.813 | 0.757 | 0.769 |
| External | 0.551 | 0.48 | 0.353 | 0.75 | 0.75 |

Table 3. Model’s performance when removing the XGBoost embeddings and reasoning module from the pipeline (direct prediction using only MOLFORMER-extracted molecular features classified via logistic regression).

| Set | AUC | F1 score | Recall | Specificity | Precision |
| --- | --- | --- | --- | --- | --- |
| Validation | 0.698 | 0.699 | 0.699 | 0.698 | 0.699 |
| Test | 0.745 | 0.748 | 0.76 | 0.730 | 0.737 |
| External | 0.397 | 0.385 | 0.294 | 0.5 | 0.556 |
